# Model-based serial blood sampling protocol for minimal mortality and better recovery in small to medium sized tilapia

**DOI:** 10.1242/bio.037978

**Published:** 2018-10-25

**Authors:** Chris Sihoka, Ina Wagenaar

**Affiliations:** Department of Zoology, University of Johannesburg, Auckland Park 2006, South Africa

**Keywords:** Serial blood sampling, Model-based, Fish

## Abstract

Serial blood sampling involving sampling blood from the same individual at different time points is essential in time-based studies including xenobiotic toxicokinetics and biochemical studies. However, high fish mortality due to phlebotomy-induced anaemia (PIA) constrains serial blood sampling in small to medium sized fish. The aim of the study was to develop and implement a model-based serial blood sampling protocol that minimises fish mortality by regulating anaemia within levels that sustain fish survival and recovery. A model simulating the reduction in haemoglobin was developed from blood sampling data of sixteen (*N*=16) medium sized *Oreochromis mossambicus*. The model was incorporated into a serial blood sampling protocol whose performance was tested on eight (*N*=8) fish. The protocol avoided fish mortality and the fish recovered from PIA within three weeks of the post-sampling period. Therefore, managing anaemia minimises mortality and improves the applicability of serial blood sampling in small to medium sized fish.

This article has an associated First Person interview with the first author of the paper..

## Abstract (precis)

**Summary:** A model-based repeated blood sampling protocol retained tolerable levels of anaemia, minimised mortality, assured recovery and made repeated blood sampling feasible in small to medium sized tilapia.

## INTRODUCTION

Fish are increasingly being used as model animals in scientific studies as they are metabolically similar to higher vertebrates in many aspects. The Mozambique tilapia (*Oreochromis mossambicus*) has several attributes which have increased its usage as a biological model. It has a rapid rate of reproduction, is euryhaline and tolerates a wider range of temperatures in the tropical and warm temperate climates (Davis et al., 2009). Blood sampling forms an important component of many studies. Serial blood sampling in which the same individual is sampled at different time points is useful in time-based studies. Time-based studies have been used to study xenobiotic toxicokinetics to understand the disposition and metabolism of a xenobiotic in the fish's body (Carbonell and Tarazona, 1994; Newby et al., 2006; Feng and Jia, 2009; Lim et al., 2010). They have also been used in biochemical studies to study fish nutrient utilization and blood acid-base state in response to stressors such as hypercapnia and hypoxia (Toews et al., 1983; Deng et al., 2000; Zang et al., 2013; Cadiz et al., 2017).

In small to medium sized fish (30–100 g tilapia) serial blood sampling is challenging to execute as smaller fish have less amounts of blood (estimated 3.9% of body weight) (Okimoto et al., 1994). Therefore, the repeated blood withdrawal often disposes the fish to severe anaemia. Anaemia reduces the supply of oxygen to the tissues leading to poor fish health (Witeska, 2015). Hence most fish fail to recover, and death often results (Department of Fisheries and Oceans, 2004). Consequently, parallel blood sampling where a different individual is sampled at each sampling time point has often been used as a substitute to serial blood sampling (Feng and Jia, 2009; Yang et al., 2014; Teles et al., 2016). Being a composite sampling technique, parallel blood sampling overlooks individual variations and therefore cannot be used in mixed effect modelling, which accurately represents toxicological processes (Tang et al., 2008; Kurawattimath et al., 2012; Sahota et al., 2015). Therefore, its application in toxicological risk assessment is limited. There is a growing advocacy for limiting the number of animals used in a scientific research (Festing and Wilkinson, 2007; Kilkenny et al., 2009). On the contrary, parallel blood sampling increases the animal sample size since a different set of individuals are sampled at each time point. This causes animal wastage on objectives that can be achieved with few animals using serial blood sampling.

Studies involving serial blood sampling are often restricted to larger fish as they have a higher blood volume and conventional blood sampling methods such as cannulation can easily be applied (Yang et al., 2014; Teles et al., 2016). A technique developed for sampling zebrafish (AB strain) with a 2.3% mortality is a rare study involving serial blood sampling in small fish (Zang et al., 2013, 2015). This study focused on the technical aspects of applying a minimally invasive blood sampling technique on zebrafish. To our knowledge, no study has attempted to model and incorporate the relationship between experimental variables (weight, sampled volume and sampling time) and a physiological parameter (blood haemoglobin concentration) responsible for fish mortality into a sampling technique. This approach offers some control on the physiological response of the fish in relation to sampling and hence can minimise mortality.

Studies have shown that repeated blood withdrawal causes a decrease in blood haemoglobin concentration in fish (Ogilvy et al., 1988; Zang et al., 2013, 2015). Haemoglobin concentration is a physiological indicator of anaemia (Witeska, 2015). Therefore, the aim of the present study was to develop and implement a model-based serial blood sampling protocol that minimises mortality of smaller fish by regulating anaemia within levels that sustain fish survival and recovery. The use of anaesthetics to sedate fish has been shown to alter haematological parameters (Smith et al., 1979; Tort et al., 2002; Trushenski et al., 2012; Gressler et al., 2014). This can confound the results in a toxicological study. Therefore, to avoid confounding errors in the model, a non-anaesthetic blood sampling technique was used.

## RESULTS AND DISCUSSION

### Model development and validation

Table 1 shows the outcome models for the mixed effect regression analysis. The model diagnostic plots for each of the tabulated models are shown in Fig. 2. Only models which accounted for interaction among variables had unbiased (homoscedastic) plots (Fig. 2A,B). All their variables [cumulative volume (V_c_), time (t), cummulative volume to mass ratio (V_c_/m)] were significant (*P*<0.01) determinants of Hb_p_ (Table 1). Conversely, models which did not account for interaction among variables produced biased plots (Fig. 2C,D) with insignificant (*P*>0.05) variable influence on Hb_p_ except for t (Table 1). Therefore, the plots showed that only models 2 and 4 were valid.

**Table 1.**
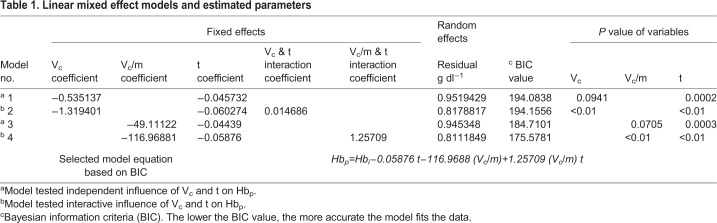
**Linear mixed effect models and estimated parameters**

**Fig. 1.**
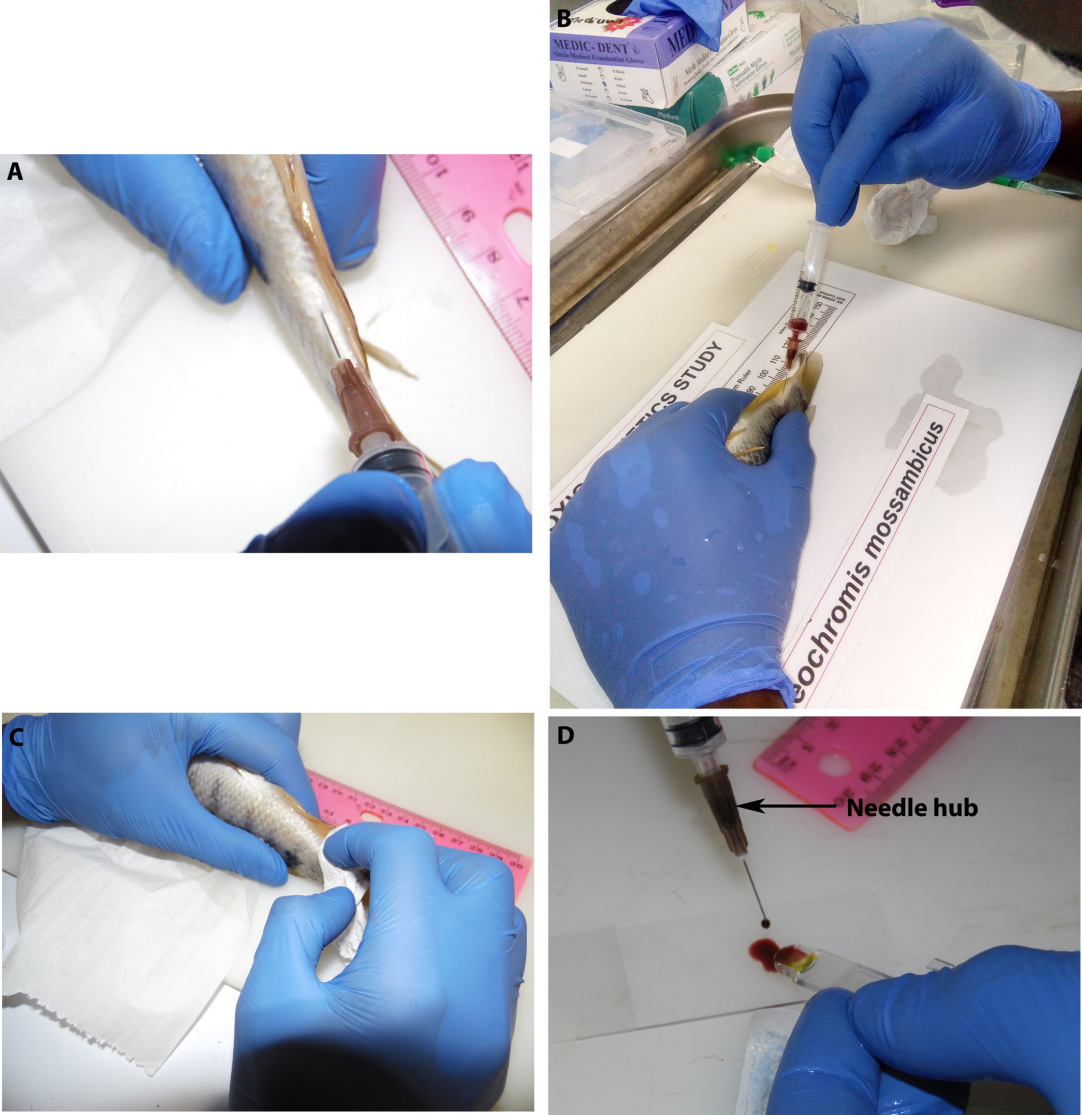
**Blood sampling and preparation for haemoglobin measurement procedure.** (A) The needle was inserted below the scales at 4–13 mm from anal fin. (B) The needle was positioned at approximately 45° ventral to caudal region and blood drawn from caudal vein. (C) A soft tissue was held over the pricked tissue for few seconds to halt the bleeding following needle withdrawal. (D) A drop of sampled blood was drawn into a Hemo Control Microcuvette in preparation for Hb measurement with a Hemo Control Hb Analyzer.

**Fig. 2.**
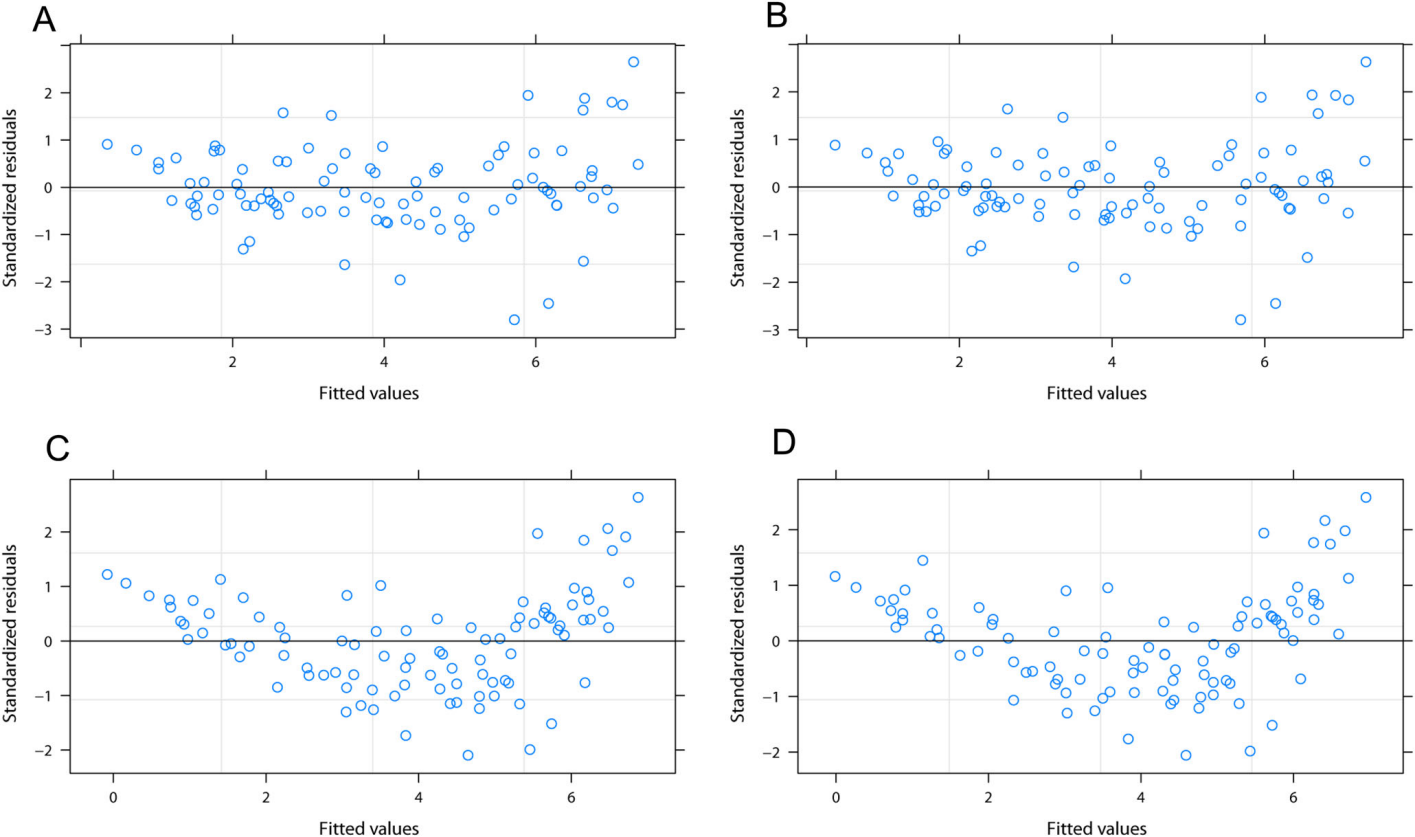
**Diagnostic plots for outcome models of linear mixed effect regression.** (A) Model 2 had a homoscedastic distribution. (B) Model 4 had a homoscedastic distribution. (C) Model 1 had a heteroscedastic distribution. (D) Model 3 had a heteroscedastic distribution.

According to the Bayesian information criterion (BIC), a model with a lower BIC value predicts a relationship better than one with a higher value (Schwarz, 1978). Model 4 had the lowest BIC value indicating that it was the best fit Model and hence was selected. This Model showed that V_c_ to m ratio (V_c_/m) and t were significant (*P*<0.01) determinants of Hb_p_. The incorporation of an interaction factor among the variables in this Model improved its performance as evidenced by a further lowering of the BIC value below that of Model 3 which did not account for interaction (Table 1). This observation strongly suggests that the parameters (Hb, V_c_, m, t) influence Hb_p_ in association rather than independently. It follows that how low Hb dropped with sampling was not only dependent on the amount of blood sampled, but also on the interval of sampling and the fish's mass.

The selected Model shows that t and V_c_ to m ratio correlated inversely with Hb_p_. This trend is consistent with what was observed in a related study on zebrafish (Zang et al., 2013). However, this study found a further association between V_c_ to m ratio and t which correlated positively with Hb_p_. The V_c_ to m ratio had the largest negative coefficient. This indicates that V_c_ to m ratio had the largest effect in reducing Hb concentration compared to other variables in the Model. Based on the Model, increasing the amount of blood sampled raises V_c_, hence increasing the V_c_ to m ratio leading to a lowering of Hb. Conversely, an increase in fish weight (m) lowers the ratio and stabilizes Hb. This phenomenon is physiologically plausible in that blood withdrawal reduces red blood cells ultimately leading to lowering of Hb (Witeska, 2015), while a larger fish is expected to have a higher total blood volume (Okimoto et al., 1994). Therefore, in a sampling exercise, optimal blood amounts ought to be drawn in relation to fish weight.

The Model accounted for approximately 0.81 g dl^−1^ residual (variation) arising from random effects which also affect Hb trend with sampling (Table 1). In this case, the most anticipated random effect is intraspecies physiological variations. It is important to note that blood withdrawal is met by various physiological responses such as those aimed at regaining optimal blood volume and osmolality. These responses could be uniquely expressed in each fish leading individual variability in Hb_p_.

Blood withdrawal in teleost species has been found to cause haemodilution due to an influx of extravascular fluid in a quest to retain optimal blood volume (Carroll et al., 1984; Ogilvy et al., 1988; Takei, 1988). A study has shown that when subjected to repeated blood withdrawal, *O. mossambicus* restores blood volume partly by drinking environmental water (Leedom et al., 2003). The haemodilution can cause a drop in Hb. The drop in Hb as represented by the Model was consistent with these physiological responses.

### Fish mortality and recovery

**Fig. 3.**
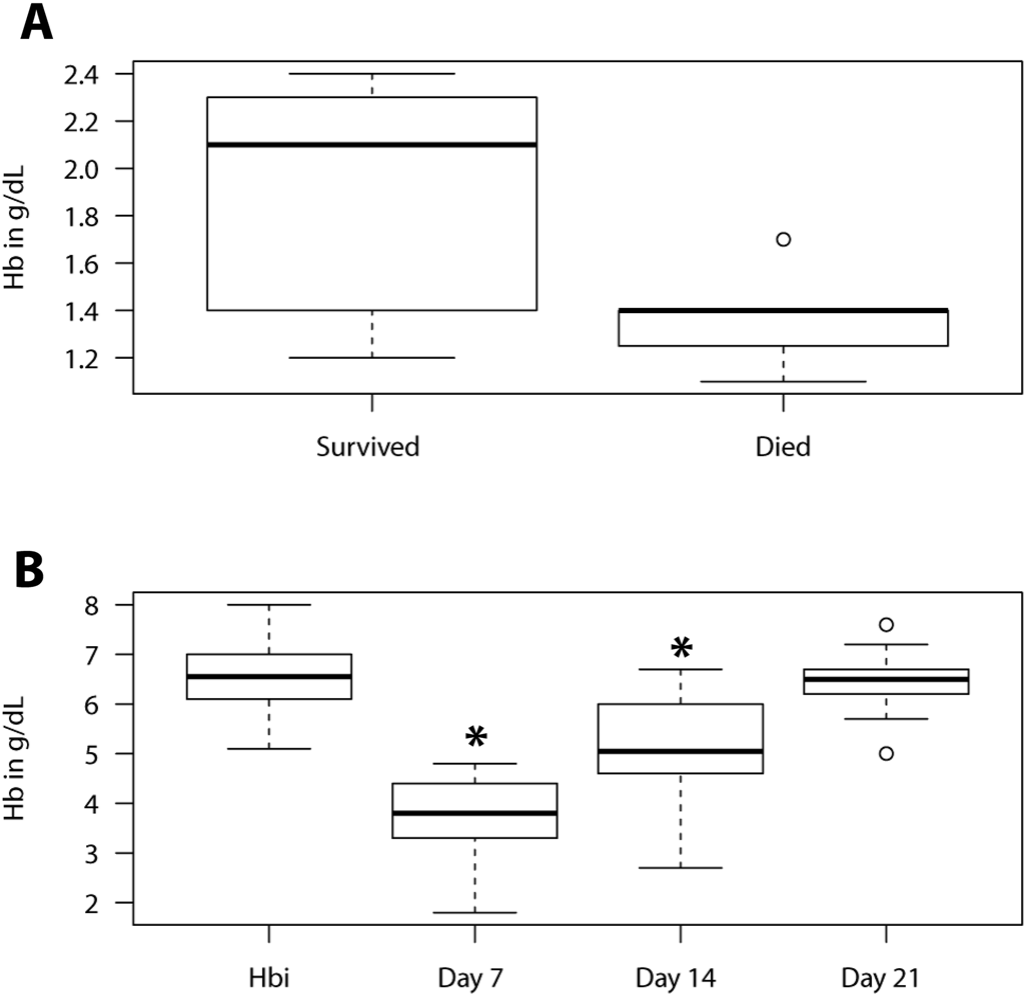
**Post-sampling haemoglobin level in the fish that died and those that survived.** (A) A boxplot showed a generally lower Hb_p_ in fish that died, 1.4 (1.3–1.4), compared to the fish that survived, 2.1 (1.6–2.3), Med (IQR) (*N*=8). (B) Kruskal–Wallis test shows that weekly Hb_p_ in surviving fish (*N*=8) increased with time and full Hb recovery was attained at 21 days as no significant difference (*P*=0.89) existed between the Hb_i_ and Hb_p_ at day 21.

Table 2 shows the odds for survival for each model variable and the mortality rate in each experiment. Haemoglobin had very high odds for survival compared to the other variables. Its odds were approximately sixfold higher than V_c_ to m ratio and eightfold higher than t. This shows that the chances of the fish surviving during sampling largely depended on the level of Hb. This notion was further complemented by a significantly lower Hb_p_ (*P*<0.05) in the fish that failed to recover (died) compared to those that survived (Fig. 3A).

**Table 2.**
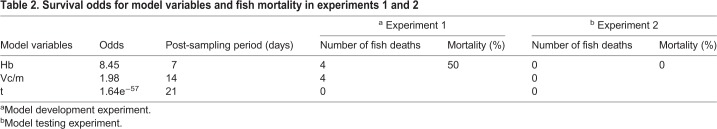
**Survival odds for model variables and fish mortality in experiments 1 and 2**

In the fish that died, the Hb_p_ dropped below 1.4 g/dl and this was significantly different from the fish that survived where Hb_p_ values were mostly above 1.4 g/dl. This trend suggests that fish recovery was immensely compromised at concentrations below 1.4 g/dl. It further affirms that fish recovery largely depended on how low the Hb level dropped. The Model-based sampling avoided fish mortality by yielding Hb_p_ concentrations that where mostly above 1.4 g/dl. We propose 1.4 g/dl as a critical threshold below which *O. mossambicus* Hb should not be allowed to drop if recovery chances are to be enhanced.

There was 50% fish mortality in the first experiment, while no mortality occurred in the Model test experiment. The mortality occurred in the first two weeks of the post-sampling period. This observation complements the findings in a related study on zebrafish where 40% mortality was recorded during a week of repeated blood sampling (Zang et al., 2013). However, mortality occurred days after sampling in our study. There was no significant variation between the Hb_i_ and Hb_p_ (*P*<0.05) by the third week (Fig. 3B). All the surviving fish had regained their normal Hb level. However, the recovery period in our study was longer than the one week found in zebrafish by Zang et al. (2013). Recovery is expected to be different in various fish species as it depends on various antianemia compensatory mechanisms which vary among species and are largely influenced by fish nutritional condition and environmental factors such as temperature and dissolved oxygen (Witeska, 2013, 2015). Lower temperature has been found to slow erythropoiesis and recovery from anaemia in *Cyprinus carpio* (Rothmann et al., 2000). Although fish may be alive a few days after sampling, our results showed that the impact of the risk imposed by the sampling on fish survival may extend for about two weeks. Therefore, we propose that fish being used for repeated blood sampling experiments should be allowed a recovery period of at least a month before being used in another blood sampling experiment.

### Model testing

**Fig. 4.**
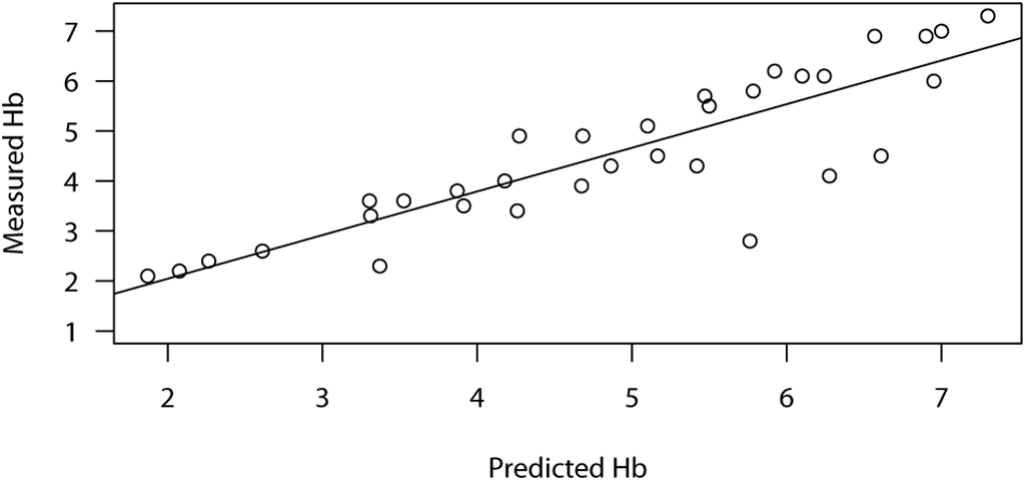
**Goodness of fit plot between model predicted and observed Hb.** There was approximately 82% (R^2^=0.8217, adjusted R^2^=0.8168) fit between the predicted and observed Hb.

Fig. 4 compares the predicted and observed Hb concentration. There was a good fit between the predicted and observed Hb as indicated by a higher coefficient of determination (R^2^=0.8217; adjusted R^2^=0.8168). This indicates that approximately 82% of the variation in Hb during sampling was accounted for by the Model. Therefore, the Model can form a reliable tool for managing Hb to tolerable limits in experimental design.

### Conclusions

Severe anaemia exemplified by a drop in Hb is among the primary causes of post sampling mortality in serially blood sampled, small to medium sized fish. A model which simulated the drop in Hb in relation to sampling experimental variables (V_c_, m and t) was developed and implemented in a serial blood sampling protocol. By regulating the Hb drop to tolerable levels the protocol minimised fish mortality. This protocol allows for less experimental animals to be used, accounts for individual variations (more accurate models) and assures recovery of sampled fish. Therefore, unnecessary killing of fish can be avoided.

## MATERIALS AND METHODS

### Experimental animals

Adult *O. mossambicus* (Peters, 1852) aged 8 months, with a mean body mass of 55.4±17.8 g and a mean standard length of 135.9±14.8 mm (mean±s.d.) were purchased from Hartbeespoort Fisheries in Johannesburg, Gauteng province, South Africa. The fish were acclimated in the University of Johannesburg Research Aquarium in an 1000 l holding tank connected to a flow-through system containing borehole water for 30 days.

### Experimental design

After the 30 day acclimation period, 24 fish (*N*=12 males, *N*=12 females) were transferred from the 1000 l holding tank to an environmental room maintained at 26±1°C with a 12 h photoperiod. The fish were divided into two groups of 16 fish (*N*=16) and 8 fish (*N*=8), with an equal number of males and females in each group. Each fish was housed in an 100 l glass tank in a flow-through system supplied with borehole water to further acclimate for 7 days (OECD, 1992; Van Dyk, et al., 2007). Fish were fed on tilapia pellets (FFTI320, Avi-products, Durban, South Africa) twice a day at about 07:30 am and 04:30 pm. Each fish was fed approximately 1% of its body weight per day. The 16 fish (*N*=16) were used for the first experiment (model development) which involved developing and validating a blood sampling model. The other fish group (*N*=8) was used for the second experiment (model testing) which involved testing the applicability of the developed model. This study was reviewed and approved by the University of Johannesburg Faculty of Science Ethics Committee (Ref. 2016-02-001).

### Model development

#### Blood sampling

An 8 inch handheld scoop net was used to pick up the fish from the water. To minimise stress caused by prolonged handling, fish were never held out of the water for a period exceeding 1 min and weighing was carried out 24 h prior to blood sampling using an electronic balance (BEB 61, BOECO, Germany). The fish were serially sampled for blood at 0, 24, 48 and 72 h time points. Blood was drawn from the caudal vein using a 0.45 mm×16 mm hypodermal needle fitted to a 2 ml graduated syringe. The needle was inserted below the scales at approximately 45° to the body axis ventral to the tail (Fig. 1A,B). The region 4 mm to 13 mm from the anal fin was used to insert the needle. A different spot was used to insert the needle at each successive sampling interval. After withdrawing the needle, a soft tissue (Wypall X50, Kimberly Clark, Bedfordview, South Africa) was held over the pricked spot for few seconds to stop the bleeding (Fig. 1C).

#### Volume and haemoglobin measurement

At each sampling time point, the volume of sampled blood was read from the graduated syringe after withdrawing the needle by holding the syringe in a vertical position. Approximately 1% body mass (0.5–1 ml) of blood was sampled at each time point. A drop of blood (approximately 50 µl) was drawn into a Hemo Control Microcuvette (REF 3000-3012-0765, EKF-diagnostic GmbH, Barleben, Germany) (Fig. 1D) to measure the blood haemoglobin concentration using a Hemo Control Analyzer (Model 3000-0031-6801, EKF diagnostic, Barleben, Germany). The sampled blood was transferred to a heparin vacutainer. The blood volume (V) sampled at each time interval (t) (0, 24, 48 and 72 h intervals) and haemoglobin (Hb) concentration were recorded. The post-sampling haemoglobin (Hb_p_) was measured 24 h after the last sampling interval by drawing a minimal volume of blood not exceeding the base of the needle hub (approximately 100 µl) (Fig. 1D).

#### Monitoring recovery

The sampled fish were maintained in their individual tanks and monitored for recovery for 3 weeks. Any fish mortalities were recorded and the percentage mortality at the end of each week was calculated as follows:
(1)
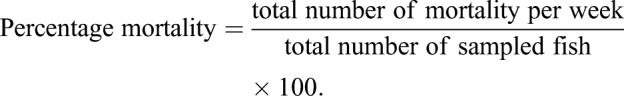
The blood Hb concentration of each fish was monitored at the end of each week. To monitor Hb, a minimal volume of blood (approximately 100 µl) was drawn as previously described.

### Statistical analysis

Statistical analyses were carried out in R environment (R statistical software version 3.3.2) at 95% confidence level. All continuous variables (m, V, Hb) were checked for normality using visual examination with Q-Q plots and the Shapiro–Wilk test.

### Modelling and model validation

Linear mixed effect regression was used to model the relationship between Hb_p_ concentration and cumulative blood volume (V_c_) sampled at each time point, with m and t as fixed effects while fish identity code as a random effect. To develop the best fit model, all variables (m, V_c_, t) were first tried as possible independent predictors (assuming no association among variables) of Hb_p_ and then as associated predictors (assuming association among variables). All models developed were examined for validity using goodness of fit plots. The best fit model was selected based on the BIC value generated by the statistical software.

### Mortality

Logistic regression was performed to determine which variables among t, Hb_p_ and V_c_/m influenced mortality and the results are reported as odds in favour of survival. The Mann–Whitney *U*-test was used to compare the Hb_p_ between the fish that survived and those that died if data were skewed and the results reported as median and interquartile range (IQR). Conversely, the unpaired *t*-test was used to compare the Hb_p_ if the data were normally distributed and results reported as mean and standard deviation (s.d.).

### Recovery

To evaluate recovery, weekly Hb_p_ were compared to the initial Hb prior to sampling (Hb_i_). The Kruskal–Wallis test was performed if data were nonparametric while the one-way ANOVA was used if data were parametric. The results are reported as median and IQR or mean and s.d., respectively.

To examine the goodness of fit between the predicted and measured Hb in the test experiment, linear regression was used to plot the predicted Hb against the measured Hb. The coefficient of determination (R^2^) is reported.

### Model testing

A minimal blood volume (approximately 2 drops) was drawn from each fish. The drawn blood was used to measure the Hb_i_ concentration of the fish as previously explained (see the volume and haemoglobin measurement section). The fish were then acclimated for 7 days. Using the measured Hb_i_, the selected Model was used to simulate two different sampling schedules lasting 24 h. In simulation, the V_c_ was adjusted to allow the predicted Hb_p_ not to fall below a critical value (median Hb_p_ of fish that died in the first experiment) and the V sampled over 24 h to be ≤1 ml. In the first sampling schedule, 0.2 ml was respectively sampled from four fish (blood sampling group 1) at 0, 2, 3, 8, and 24 h time points. In the second sampling schedule 0.6 ml was respectively sampled from the other four fish (blood sampling group 2) at 0, and 24 h time points. The sampled fish were monitored for recovery for 3 weeks. Fish mortality and Hb concentration were monitored as previously explained (see the monitoring recovery section of the Materials and Methods).
